# Supplementary material for: Learning strategies and long-term memory in Asian short-clawed otters (Aonyx cinereus)
Source: R Soc Open Sci. 2020 Nov 11;7(11):201215. doi: 10.1098/rsos.201215 (PMC7735368; doi:10.1098/rsos.201215)
Supplement: Supplementary Materials [file rsos201215supp1.docx]

**Appendix/Supplementary Material**

**1. Notes relating to network-based diffusion analysis (NBDA)**

*1.1 The effect on a group’s association network of temporarily removing and re-adding otters*

Although the removal and reintroduction of the unwell otter may have modified the original pre-removal five-otter network for the New Forest group, the fact that the NBDA run using the original network provided significant evidence for social transmission (see Results) suggests that this network was still representative of the group’s social structure. If this were not the case, we would not expect to find any correlation between the network and the order of information acquisition. More generally, the more ‘noise’ the networks contain, the more conservative estimates of social transmission become. Thus, the detection of patterns in the data consistent with social transmission following the network suggest that the networks are meaningful in that they approximate the pathways of transmission.

*1.2 Pseudoreplication in NBDA*

A potential criticism of NBDA is that it is for possible a type 1 error to occur if individuals learn new behaviours asocially in an order that follows the strength of associations in the network. This possibility is assessed by, and reflected in, the confidence intervals for the social transmission parameter estimates and AIC values, on the assumption that each diffusion has different individuals. Here, there were a total of five diffusions per otter group (one diffusion for each of the different task types). So if otters in each of the three groups asocially learned to interact with, and solve, tasks in the same characteristic order in each diffusion, and this order also happened to follow the network, then the evidence for social learning could have been inflated. However this was not the case here, as otters in each group tended to learn to interact with, and solve, tasks in a different order in each diffusion (data available through the Dryad Digital Repository: doi:10.5061/dryad.sf7m0cg3s). Thus, learning order is unlikely to have affected the evidence presented for social learning.

**2. Tables**

**Table S1.** Group compositions for each otter group in this study, and the characteristics of every individual in those groups. Individual characteristics include: the identification (ID) number assigned to each individual for data analyses, sex, age, and whether they were a parent or offspring. Age was quantified in years, sex was either male (M) or female (F), and individuals were either parents (Y) or offspring (N). For individuals less than a year old, their ages in months (to the nearest annual quarter) are reported as proportions of year for both the first and second rounds of task presentations.

| **Wildlife Centre** | **Network ID** | **Name** | **Sex** | **Age (years)** | **Parent** | **Social relationship** | **Social Network** |
| --- | --- | --- | --- | --- | --- | --- | --- |
| Newquay Zoo | 1 | Tope | M | 9 | Y | Father | Network 1 & 2 |
| Newquay Zoo | 2 | Jam | F | 7 | Y | Mother | Network 1 & 2 |
| Newquay Zoo | 3 | Pod | F | 4 | N | Litter 1 | Network 1 & 2 |
| Newquay Zoo | 4 | Meg | F | 1 | N | Litter 2 | Network 1 & 2 |
| Newquay Zoo | 5 | Leo | M | 1 | N | Litter 2 | Network 1 |
| Newquay Zoo | 6 | Milo | M | 1 | N | Litter 2 | Network 1 |
| Newquay Zoo | 7 | Geoff | M | 1 | N | Litter 2 | Network 1 |
| Newquay Zoo | 8 | Rosie | F | 0.25/0.75 | N | Litter 3 | Network 1 & 2 |
| Newquay Zoo | 9 | Dot | F | 0.25/0.75 | N | Litter 3 | Network 1 & 2 |
| Newquay Zoo | 10 | Charlie | M | 0.25/0.75 | N | Litter 3 | Network 1 & 2 |
| Newquay Zoo | 11 | Biscuit | F | 0.25 | N | Litter 3 | Network 1 |
| Newquay Zoo | 12 | Pepe | M | 0.25/0.75 | N | Litter 3 | Network 1 & 2 |
| Tamar Otter and Wildlife Centre | 1 | Leah | F | 10 | Y | Mother | Network 1 |
| Tamar Otter and Wildlife Centre | 2 | Feet | M | 9 | Y | Father | Network 1 |
| Tamar Otter and Wildlife Centre | 3 | India | F | 5 | N | Litter 1 | Network 1 |
| Tamar Otter and Wildlife Centre | 4 | Chai | M | 5 | N | Litter 1 | Network 1 |
| Tamar Otter and Wildlife Centre | 5 | Cassia | M | 3 | N | Litter 2 | Network 1 |
| Tamar Otter and Wildlife Centre | 6 | Cameron | M | 3 | N | Litter 2 | Network 1 |
| Tamar Otter and Wildlife Centre | 7 | Hazel | F | 1 | N | Litter 3 | Network 1 |
| Tamar Otter and Wildlife Centre | 8 | Daisy | F | 1 | N | Litter 3 | Network 1 |
| Tamar Otter and Wildlife Centre | 9 | Harry | M | 1 | N | Litter 3 | Network 1 |
| Tamar Otter and Wildlife Centre | 10 | Dougie | M | 1 | N | Litter 3 | Network 1 |
| Tamar Otter and Wildlife Centre | 11 | Rani | F | 0.5/0.75 | N | Litter 4 | Network 1 |
| Tamar Otter and Wildlife Centre | 12 | Khan | M | 0.5/0.75 | N | Litter 4 | Network 1 |
| New Forest Wildlife Park | 1 | Flint | M | 13 | N | Litter 1 | Network 1 & 2 |
| New Forest Wildlife Park | 2 | Magma | M | 11 | N | Litter 3 | Network 1 & 2 |
| New Forest Wildlife Park | 3 | Fossil | M | 11 | N | Litter 3 | Network 1 & 2 |
| New Forest Wildlife Park | 4 | Earth | F | 12 | N | Litter 2 | Network 1 & 2 |
| New Forest Wildlife Park | 5 | Moon | F | 12 | N | Litter 2 | Network 1 |

**Table S2.** Task dimensions, and details of how the food rewards were accessed from each task type.

| **Task type** | **Dimensions (mm)** | **Description** |
| --- | --- | --- |
| 1 | 130 x 70 x 70 | A plastic cup with hay covering the opening, otters had to remove the hay to access the food reward. |
| 2 | 110 x 180 x 70 | A glued-shut plastic Tupperware box containing a divider, with a hole in the lid either side of the divider. Otters had to correctly identify the side containing the food reward and reach through the corresponding hole to retrieve it. |
| 3 | 75 x 105 x 80 | A plastic Tupperware box with three of the four lid clips removed, and the fourth lid clip glued to the box to make a flap opening. Otters had to lift the flap to access the food reward. |
| 4 | 150 x 150 x 105 | A bowl-shaped plastic Tupperware, with an opening cut into the bottom, inverted to create a dome, and fixed to a dowel to facilitate rotation. The food reward was placed behind a vertical separator inside the dome, and otters had to rotate the dome to align the opening with the food reward. |
| 5 | 85 x 110 x 180 | Inverted plastic Tupperware box with the lid glued shut and an opening cut into one side. A pull tab, on which the food reward rested, was inserted above the opening, and otters had to pull the tab to drop the food reward into the open section. |

*Each task apparatus was fixed to a piece of medium-density fibreboard (300 x 300 x 12mm for task types 1, 3, 4, and 5, and 400 x 300 x 12 mm for task type 2) to prevent the otters from moving them around the enclosure.

**Table S3.** The number of incidences in which otters from each group were first to interact with and/or first solve a task type, as well as the task types otters interacted with or solved during the course of task presentation round 1. In some cases, the first interaction event was tied in time between two or more individuals.

| **Wildlife centre** | **Name** | **Incidences of first interaction** | **Incidences of first solve** | **Task types interacted with** | **Task types solved** |
| --- | --- | --- | --- | --- | --- |
| Newquay Zoo | Tope | 2 | 2 | 1, 2, 3, 4, 5 | 1, 2, 3, 4, 5 |
| Newquay Zoo | Jam | 2 | 1 | 1, 2, 3, 4, 5 | 1, 2, 3, 4, 5 |
| Newquay Zoo | Pod | 0 | 0 | 1, 2, 3, 4, 5 | 1, 2 |
| Newquay Zoo | Meg | 1 | 1 | 1, 2, 3, 4, 5 | 1, 3, 4, 5 |
| Newquay Zoo | Leo | 1 | 0 | 1, 2, 3, 4, 5 | 1, 4, 5 |
| Newquay Zoo | Milo | 1 | 0 | 1, 2, 3, 4, 5 | 1, 2, 3, 4 |
| Newquay Zoo | Geoff | 0 | 0 | 1, 2, 3, 4, 5 | 2, 3, 4 |
| Newquay Zoo | Rosie | 0 | 0 | 2, 3, 4, 5 | None |
| Newquay Zoo | Dot | 0 | 0 | 2, 3, 5 | None |
| Newquay Zoo | Charlie | 0 | 0 | 2, 3, 4, 5 | None |
| Newquay Zoo | Biscuit | 0 | 0 | 2, 5 | None |
| Newquay Zoo | Pepe | 0 | 0 | 3, 5 | None |
| Tamar Otter and Wildlife Centre | Leah | 4 | 5 | 1, 2, 3, 4, 5 | 1, 2, 3, 4, 5 |
| Tamar Otter and Wildlife Centre | Feet | 0 | 0 | 3, 4, 5 | None |
| Tamar Otter and Wildlife Centre | India | 0 | 0 | 3, 4, 5 | 3, 4, 5 |
| Tamar Otter and Wildlife Centre | Chai | 0 | 0 | 1, 2, 3, 4, 5 | 1, 2, 4, 5 |
| Tamar Otter and Wildlife Centre | Cassia | 0 | 0 | 1, 2, 3, 4, 5 | 1, 2, 4 |
| Tamar Otter and Wildlife Centre | Cameron | 0 | 0 | 1, 2, 3, 4, 5 | 1, 2, 3, 4, 5 |
| Tamar Otter and Wildlife Centre | Hazel | 0 | 0 | 3, 4, 5 | 3 |
| Tamar Otter and Wildlife Centre | Daisy | 0 | 0 | 2, 3, 4, 5 | None |
| Tamar Otter and Wildlife Centre | Harry | 1 | 0 | 1, 2, 3, 4, 5 | 1, 2, 3, 4, 5 |
| Tamar Otter and Wildlife Centre | Dougie | 0 | 0 | 1, 3, 4, 5 | 1, 3, 5 |
| Tamar Otter and Wildlife Centre | Rani | 0 | 0 | 2, 3, 4, 5 | None |
| Tamar Otter and Wildlife Centre | Khan | 0 | 0 | 1, 3, 4, 5 | None |
| New Forest Wildlife Park | Flint | 5 | 0 | 1, 2, 3, 4, 5 | 1, 2, 4 |
| New Forest Wildlife Park | Magma | 2 | 2 | 1, 2, 3, 4, 5 | 1, 2, 3, 4, 5 |
| New Forest Wildlife Park | Fossil | 5 | 3 | 1, 2, 3, 4, 5 | 1, 2, 3, 4 |
| New Forest Wildlife Park | Earth | 1 | 0 | 1, 3, 4, 5 | 1, 3 |

**Table S4.** Model selection table for the TADA analysing the time sequence in which otters first interacted with tasks in the first round of task presentations. Models depicted are the best-supported models of each type, with each type of baseline, fit to each network type. Findings from the top ranked model (*italicised* and highlighted in **bold**) are reported in the text.

| **Type** | **Rate of social transmission between tasks** | **Baseline rate** | **Network** | | **ILVs** | **logLik** | **AIC** | **Δ AIC** | **Weight** |
| --- | --- | --- | --- | --- | --- | --- | --- | --- | --- |
| ***Multiplicative*** | ***Same*** | ***Gamma*** | ***Social*** | ***Age*** | | ***672.05*** | ***1352.09*** | ***0.00*** | ***0.45*** |
| Multiplicative | Same | Gamma | Group | Age | | 673.05 | 1354.11 | 2.02 | 0.16 |
| Multiplicative | Different | Gamma | Social | Age | | 670.12 | 1356.24 | 4.15 | 0.03 |
| Multiplicative | Different | Gamma | Group | Age | | 670.78 | 1357.56 | 5.47 | 0.02 |
| Additive | Same | Gamma | Social | Age | | 675.94 | 1359.88 | 7.79 | 0.01 |
| Additive | Different | Gamma | Social | Age | | 672.92 | 1361.85 | 9.76 | < 0.01 |
| Additive | Different | Gamma | Group | Age | | 674.35 | 1364.70 | 12.61 | < 0.01 |
| Additive | Same | Gamma | Group | Age | | 678.83 | 1365.65 | 13.56 | < 0.01 |
| No ILVs | Same | Gamma | Social | None | | 692.85 | 1391.71 | 39.62 | < 0.01 |
| No ILVs | Different | Gamma | Social | None | | 690.01 | 1394.01 | 41.92 | < 0.01 |
| Asocial | None | Gamma | Asocial | Age | | 696.04 | 1398.09 | 46.00 | < 0.01 |
| No ILVs | Different | Gamma | Group | None | | 693.25 | 1400.51 | 48.42 | < 0.01 |
| No ILVs | Same | Gamma | Group | None | | 697.67 | 1401.34 | 49.25 | < 0.01 |
| Additive | Different | Constant | Group | Age | | 700.39 | 1414.78 | 62.69 | < 0.01 |
| Additive | Different | Constant | Social | Age | | 701.29 | 1416.59 | 64.50 | < 0.01 |
| Multiplicative | Different | Constant | Group | Age | | 702.64 | 1419.28 | 67.19 | < 0.01 |
| Multiplicative | Different | Constant | Social | Age | | 703.71 | 1421.41 | 69.32 | < 0.01 |
| Multiplicative | Same | Constant | Group | Age | | 711.23 | 1428.48 | 76.39 | < 0.01 |
| Multiplicative | Same | Constant | Social | Age | | 711.36 | 1428.73 | 76.64 | < 0.01 |
| Additive | Same | Constant | Social | Age | | 711.44 | 1428.88 | 76.79 | < 0.01 |
| Asocial | None | Constant | Asocial | Age | | 712.56 | 1429.13 | 77.04 | < 0.01 |
| Additive | Same | Constant | Group | Age | | 711.64 | 1429.27 | 77.18 | < 0.01 |
| No ILVs | Different | Constant | Group | None | | 723.43 | 1458.88 | 106.79 | < 0.01 |
| No ILVs | Different | Constant | Social | None | | 723.90 | 1459.81 | 107.72 | < 0.01 |
| No ILVs | Same | Constant | Group | None | | 733.36 | 1470.73 | 118.64 | < 0.01 |
| No ILVs | Same | Constant | Social | None | | 733.36 | 1470.73 | 118.64 | < 0.01 |

**Table S5.** Model selection table for the TADA analysing the time sequence in which otters first solved tasks in the first round of task presentations. Models depicted are the best-supported models of each type, with each type of baseline, fit to each network type. Findings from the top ranked model (*italicised* and highlighted in **bold**) are reported in the text.

| **Type** | **Rate of social transmission between tasks** | **Baseline rate** | **Network** | | **ILVs** | **logLik** | **AIC** | **Δ AIC** | **Weight** |
| --- | --- | --- | --- | --- | --- | --- | --- | --- | --- |
| ***Multiplicative*** | ***Different*** | ***Gamma*** | ***Social*** | ***Age, Sex*** | | ***501.89*** | ***1024.88*** | ***0.00*** | ***0.48*** |
| Multiplicative | Different | Gamma | Group | Age, Sex | | 503.89 | 1028.88 | 4.00 | 0.06 |
| Multiplicative | Same | Gamma | Social | Age, Sex | | 509.93 | 1030.83 | 5.95 | 0.02 |
| Additive | Different | Gamma | Social | Age | | 506.42 | 1031.28 | 6.40 | 0.02 |
| Additive | Same | Gamma | Social | Age | | 512.13 | 1032.91 | 8.03 | 0.01 |
| Multiplicative | Same | Gamma | Group | Age, Sex | | 511.55 | 1034.08 | 9.20 | 0.01 |
| Asocial | None | Gamma | Asocial | Age | | 514.08 | 1036.81 | 11.93 | < 0.01 |
| Additive | Same | Gamma | Group | Age | | 514.56 | 1037.75 | 12.87 | < 0.01 |
| Additive | Different | Gamma | Group | Age | | 508.57 | 1038.24 | 13.36 | < 0.01 |
| No ILVs | Different | Gamma | Social | None | | 516.61 | 1049.08 | 24.20 | < 0.01 |
| Multiplicative | Different | Constant | Social | Age, Sex | | 516.49 | 1051.41 | 26.53 | < 0.01 |
| No ILVs | Different | Gamma | Group | None | | 518.99 | 1053.78 | 28.90 | < 0.01 |
| No ILVs | Same | Gamma | Social | None | | 523.88 | 1054.13 | 29.25 | < 0.01 |
| Multiplicative | Different | Constant | Group | Age, Sex | | 518.13 | 1054.71 | 29.83 | < 0.01 |
| No ILVs | Same | Gamma | Group | None | | 524.58 | 1055.54 | 30.66 | < 0.01 |
| Additive | Different | Constant | Social | Age | | 521.19 | 1058.24 | 33.36 | < 0.01 |
| Additive | Different | Constant | Group | Age | | 522.69 | 1061.24 | 36.36 | < 0.01 |
| Asocial | None | Constant | Asocial | Age | | 528.12 | 1062.61 | 37.73 | < 0.01 |
| Multiplicative | Same | Constant | Group | Age, Sex | | 528.12 | 1064.87 | 39.99 | < 0.01 |
| Multiplicative | Same | Constant | Social | Age, Sex | | 528.12 | 1064.87 | 39.99 | < 0.01 |
| Additive | Same | Constant | Social | Age | | 528.12 | 1064.87 | 39.99 | < 0.01 |
| Additive | Same | Constant | Group | Age | | 528.12 | 1064.87 | 39.99 | < 0.01 |
| No ILVs | Different | Constant | Social | None | | 528.26 | 1069.89 | 45.01 | < 0.01 |
| No ILVs | Different | Constant | Group | None | | 530.08 | 1073.54 | 48.66 | < 0.01 |
| No ILVs | Same | Constant | Group | None | | 536.72 | 1077.63 | 52.75 | < 0.01 |
| No ILVs | Same | Constant | Social | None | | 536.72 | 1077.63 | 52.75 | < 0.01 |

**Table S6.** The number of incidences in which otters from each group were first to interact with and/or first solve a task type, as well as the task types otters interacted with or solved during the course of task presentation round 2. In some cases, the first interaction event was tied in time between two or more individuals.

| **Wildlife centre** | **Name** | **Incidences of first interaction** | **Incidences of first solve** | **Task types interacted with** | **Task types solved** |
| --- | --- | --- | --- | --- | --- |
| Newquay Zoo | Tope | 2 | 3 | 1, 2, 3, 4, 5 | 1, 2, 3, 4, 5 |
| Newquay Zoo | Jam | 1 | 1 | 1, 2, 3, 4, 5 | 1, 2, 3, 4, 5 |
| Newquay Zoo | Pod | 1 | 0 | 1, 2, 3, 4, 5 | 1, 2, 5 |
| Newquay Zoo | Meg | 1 | 0 | 1, 2, 3, 4, 5 | 2, 3, 4 |
| Newquay Zoo | Rosie | 0 | 0 | 2, 3, 4, 5 | None |
| Newquay Zoo | Dot | 0 | 1 | 1, 2, 3, 4, 5 | 2, 5 |
| Newquay Zoo | Charlie | 0 | 0 | 2, 3, 4, 5 | None |
| Newquay Zoo | Pepe | 0 | 0 | 2, 3, 4, 5 | None |
| Tamar Otter and Wildlife Centre | Leah | 2 | 3 | 1, 2, 3, 4, 5 | 1, 2, 3, 4, 5 |
| Tamar Otter and Wildlife Centre | Feet | 0 | 0 | 1, 3, 4, 5 | None |
| Tamar Otter and Wildlife Centre | India | 1 | 0 | 1, 2, 3, 4, 5 | 1, 2, 3, 4, 5 |
| Tamar Otter and Wildlife Centre | Chai | 2 | 1 | 1, 2, 3, 4, 5 | 1, 2, 4, 5 |
| Tamar Otter and Wildlife Centre | Cassia | 1 | 0 | 2, 3, 4, 5 | 2, 4 |
| Tamar Otter and Wildlife Centre | Cameron | 2 | 0 | 1, 2, 3, 4, 5 | 1, 2, 3, 5 |
| Tamar Otter and Wildlife Centre | Hazel | 1 | 0 | 2, 3, 4, 5 | 2, 3 |
| Tamar Otter and Wildlife Centre | Daisy | 0 | 0 | 3, 4, 5 | None |
| Tamar Otter and Wildlife Centre | Harry | 0 | 2 | 1, 2, 3, 4, 5 | 1, 2, 3, 5 |
| Tamar Otter and Wildlife Centre | Dougie | 0 | 0 | 3, 4, 5 | 5 |
| Tamar Otter and Wildlife Centre | Rani | 0 | 0 | 3, 4, 5 | None |
| Tamar Otter and Wildlife Centre | Khan | 0 | 0 | 3, 4, 5 | None |
| New Forest Wildlife Park | Flint | 3 | 0 | 1, 2, 3, 4, 5 | 1, 2, 3, 4 |
| New Forest Wildlife Park | Magma | 2 | 5 | 1, 2, 3, 4, 5 | 1, 2, 3, 4, 5 |
| New Forest Wildlife Park | Fossil | 1 | 0 | 1, 2, 3, 4, 5 | 2, 3 |
| New Forest Wildlife Park | Earth | 0 | 0 | 1, 2, 3, 4, 5 | 1 |
| New Forest Wildlife Park | Moon | 0 | 0 | 1, 2, 3, 4, 5 | 1, 2, 3 |

**Table S7.** Model selection table for the TADA analysing the time sequence in which otters first interacted with tasks in the second round of task presentations. Models depicted are the best-supported models of each type, with each type of baseline, fit to each network type. Findings from the top ranked model (*italicised* and highlighted in **bold**) are reported in the text.

| **Type** | **Rate of social transmission between tasks** | **Baseline rate** | **Network** | | **ILVs** | **logLik** | **AIC** | **Δ AIC** | **Weight** |
| --- | --- | --- | --- | --- | --- | --- | --- | --- | --- |
| ***Multiplicative*** | ***Different*** | ***Gamma*** | ***Social*** | ***Age*** | | ***532.86*** | ***1081.71*** | ***0.00*** | ***0.61*** |
| Multiplicative | Different | Gamma | Group | Age | | 535.36 | 1086.72 | 5.01 | 0.05 |
| Multiplicative | Different | Constant | Social | Age | | 537.41 | 1088.83 | 7.12 | 0.02 |
| Additive | Different | Gamma | Social | Age | | 537.58 | 1091.17 | 9.46 | 0.01 |
| Multiplicative | Different | Constant | Group | Age | | 539.92 | 1093.84 | 12.13 | < 0.01 |
| Additive | Different | Constant | Social | Age | | 540.52 | 1095.05 | 13.34 | < 0.01 |
| Multiplicative | Same | Gamma | Social | Age | | 544.42 | 1096.84 | 15.13 | < 0.01 |
| Additive | Different | Gamma | Group | Age | | 541.23 | 1098.45 | 16.74 | < 0.01 |
| No ILVs | Different | Gamma | Social | None | | 542.42 | 1098.85 | 17.14 | < 0.01 |
| Multiplicative | Same | Gamma | Group | Age | | 546.20 | 1100.40 | 18.69 | < 0.01 |
| Additive | Different | Constant | Group | Age | | 544.00 | 1102.00 | 20.29 | < 0.01 |
| No ILVs | Different | Constant | Social | None | | 545.92 | 1103.84 | 22.13 | < 0.01 |
| No ILVs | Different | Gamma | Group | None | | 545.94 | 1105.89 | 24.18 | < 0.01 |
| No ILVs | Different | Constant | Group | None | | 549.21 | 1110.42 | 28.71 | < 0.01 |
| Additive | Same | Gamma | Social | Age | | 555.81 | 1119.62 | 37.91 | < 0.01 |
| Multiplicative | Same | Constant | Social | Age | | 558.23 | 1122.47 | 40.76 | < 0.01 |
| Multiplicative | Same | Constant | Group | Age | | 558.74 | 1123.48 | 41.77 | < 0.01 |
| Additive | Same | Gamma | Group | Age | | 558.31 | 1124.62 | 42.91 | < 0.01 |
| No ILVs | Same | Gamma | Social | None | | 560.63 | 1127.26 | 45.55 | < 0.01 |
| No ILVs | Same | Gamma | Group | None | | 562.88 | 1131.75 | 50.04 | < 0.01 |
| Additive | Same | Constant | Social | Age | | 564.17 | 1134.35 | 52.64 | < 0.01 |
| Additive | Same | Constant | Group | Age | | 564.71 | 1135.41 | 53.70 | < 0.01 |
| No ILVs | Same | Constant | Group | None | | 567.88 | 1139.76 | 58.05 | < 0.01 |
| No ILVs | Same | Constant | Group | None | | 568.28 | 1140.56 | 58.85 | < 0.01 |
| Asocial | None | Constant | Asocial | Age | | 569.63 | 1143.26 | 61.55 | < 0.01 |
| Asocial | None | Gamma | Asocial | Age | | 569.59 | 1145.18 | 63.47 | < 0.01 |

**Table S8.** Model selection table for the TADA analysing the time sequence in which otters first solved tasks in the second round of task presentations. Models depicted are the best-supported models of each type, with each type of baseline, fit to each network type. Findings from the top ranked model (*italicised* and highlighted in **bold**) are reported in the text.

| **Type** | **Rate of social transmission between tasks** | **Baseline rate** | **Network** | | **ILVs** | **logLik** | **AIC** | **Δ AIC** | **Weight** |
| --- | --- | --- | --- | --- | --- | --- | --- | --- | --- |
| ***Multiplicative*** | ***Different*** | ***Gamma*** | ***Social*** | ***Age*** | | ***407.43*** | ***830.86*** | ***0.00*** | ***0.27*** |
| Multiplicative | Different | Gamma | Group | Age | | 407.78 | 831.58 | 0.72 | 0.19 |
| Asocial | None | Gamma | Asocial | Age | | 415.06 | 836.11 | 5.25 | 0.06 |
| Additive | Different | Gamma | Social | Age | | 410.32 | 836.64 | 5.78 | 0.01 |
| Additive | Different | Gamma | Group | Age | | 410.43 | 836.86 | 6.00 | 0.01 |
| Multiplicative | Same | Gamma | Social | Age | | 414.95 | 837.90 | 7.04 | 0.02 |
| Multiplicative | Same | Gamma | Group | Age | | 414.99 | 837.97 | 7.11 | 0.02 |
| Additive | Same | Gamma | Group | Age | | 415.06 | 838.11 | 7.25 | 0.02 |
| Additive | Same | Gamma | Social | Age | | 415.06 | 838.11 | 7.78 | 0.02 |
| Multiplicative | Different | Constant | Social | Age, Sex | | 411.52 | 839.05 | 8.19 | < 0.01 |
| Multiplicative | Different | Constant | Group | Age, Sex | | 411.71 | 839.42 | 8.56 | < 0.01 |
| Asocial | None | Constant | Asocial | Age | | 418.36 | 842.73 | 11.87 | < 0.01 |
| Additive | Different | Constant | Social | Age | | 413.79 | 843.58 | 12.72 | < 0.01 |
| Additive | Different | Constant | Group | Age | | 413.81 | 843.62 | 12.76 | < 0.01 |
| Additive | Same | Constant | Group | Age | | 418.36 | 844.73 | 13.87 | < 0.01 |
| Multiplicative | Same | Constant | Group | Age, Sex | | 418.36 | 844.73 | 13.87 | < 0.01 |
| Multiplicative | Same | Constant | Social | Age, Sex | | 418.36 | 844.73 | 13.87 | < 0.01 |
| Additive | Same | Constant | Social | Age | | 418.36 | 844.73 | 13.87 | < 0.01 |
| No ILVs | Different | Gamma | Social | None | | 418.66 | 851.33 | 20.47 | < 0.01 |
| No ILVs | Different | Gamma | Group | None | | 418.83 | 851.65 | 20.79 | < 0.01 |
| No ILVs | Same | Gamma | Group | None | | 423.82 | 853.65 | 22.79 | < 0.01 |
| No ILVs | Same | Gamma | Social | None | | 423.82 | 853.65 | 22.79 | < 0.01 |
| No ILVs | Different | Constant | Social | None | | 424.76 | 861.53 | 30.67 | < 0.01 |
| No ILVs | Different | Constant | Group | None | | 424.79 | 861.59 | 30.73 | < 0.01 |
| No ILVs | Same | Constant | Group | None | | 429.38 | 862.77 | 31.91 | < 0.01 |
| No ILVs | Same | Constant | Social | None | | 429.38 | 862.77 | 31.91 | < 0.01 |

**Table S9.** A comparison of the relative support (*i.e.* percentage of overall support based on summed Akaike weights) for different social and asocial learning models with constant and gamma baselines, fit to group (homogeneous associations between individuals) and social (heterogenous associations between individuals) networks, for the first instance each otter interacted with, and solved, novel foraging tasks during each round of task presentations. *Italicised* values in **bold** indicate the model types with the most statistical support for each information type in each presentation round.

|  |  | **Round 1** | | **Round 2** | |
| --- | --- | --- | --- | --- | --- |
| **Model type** | **Rate of social transmission**  **between tasks** | **Interaction** | **Solve** | **Interaction** | **Solve** |
| **Constant baseline** |  |  |  |  |  |
| Asocial |  | < 0.01 | < 0.01 | < 0.01 | 0.45 |
| Group network |  |  |  |  |  |
| Additive | Same | < 0.01 | < 0.01 | < 0.01 | 0.15 |
|  | Different | < 0.01 | < 0.01 | < 0.01 | 0.09 |
| Multiplicative | Same | < 0.01 | < 0.01 | < 0.01 | 0.15 |
|  | Different | < 0.01 | < 0.01 | 0.23 | 0.67 |
| No ILVs | Same | < 0.01 | < 0.01 | < 0.01 | < 0.01 |
|  | Different | < 0.01 | < 0.01 | < 0.01 | <0.01 |
| Social network |  |  |  |  |  |
| Additive | Same | < 0.01 | < 0.01 | < 0.01 | 0.15 |
|  | Different | < 0.01 | < 0.01 | 0.13 | 0.09 |
| Multiplicative | Same | < 0.01 | < 0.01 | < 0.01 | 0.15 |
|  | Different | < 0.01 | < 0.01 | 2.84 | 0.80 |
| No ILVs | Same | < 0.01 | < 0.01 | < 0.01 | <0.01 |
|  | Different | < 0.01 | < 0.01 | < 0.01 | <0.01 |
| **Gamma baseline** |  |  |  |  |  |
| Asocial |  | < 0.01 | 0.24 | < 0.01 | 10.09 |
| Group network |  |  |  |  |  |
| Additive | Same | 0.07 | 1.21 | < 0.01 | 3.15 |
|  | Different | 0.05 | 0.06 | < 0.01 | 2.02 |
| Multiplicative | Same | 23.94 | 0.85 | 0.01 | 3.31 |
|  | Different | 2.47 | 10.76 | 7.26 | 28.83 |
| No ILVs | Same | < 0.01 | < 0.01 | < 0.01 | <0.01 |
|  | Different | < 0.01 | < 0.01 | < 0.01 | <0.01 |
| Social Network |  |  |  |  |  |
| Additive | Same | 1.25 | 1.15 | < 0.01 | 3.15 |
|  | Different | 0.24 | 2.54 | 0.74 | 2.26 |
| Multiplicative | Same | ***67.13*** | 4.51 | 0.07 | 3.40 |
|  | Different | 4.84 | ***79.77*** | ***88.68*** | ***41.09*** |
| No ILVs | Same | < 0.01 | < 0.01 | < 0.01 | <0.01 |
|  | Different | < 0.01 | < 0.01 | 0.01 | <0.01 |

**3. Figures**


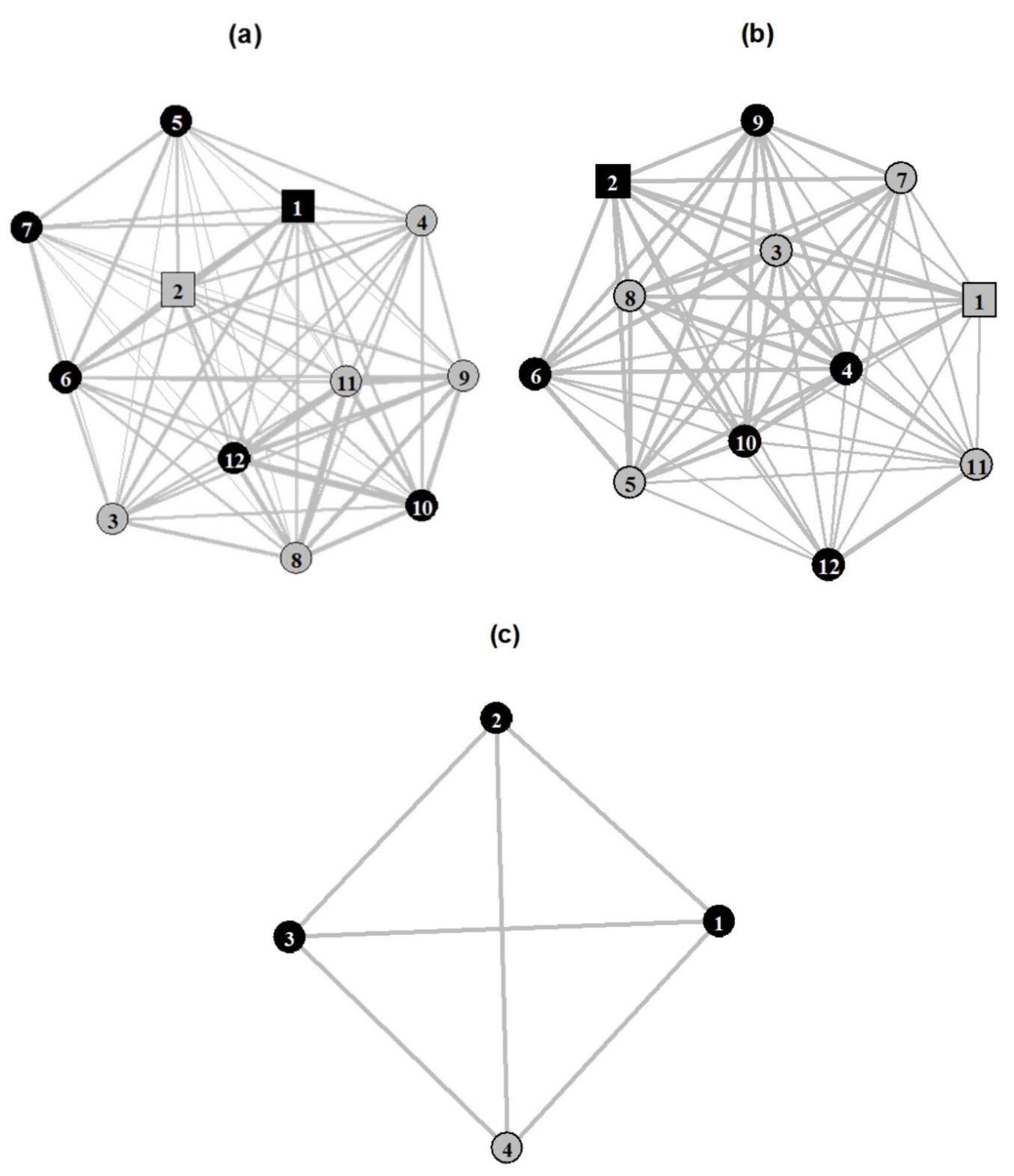


**Figure S1.** The Association Networks of the Asian short-clawed otter groups during the first round of the novel foraging task presentations; (a) Newquay Zoo (social differentiation coefficient; SDC: 0.48), (b) Tamar Otter and Wildlife Centre (SDC: 0.36), and (c) New Forest Wildlife Park (SDC: 0.52). Nodes represent group members; squares and circles represent parents and offspring respectively, and black and grey nodes represent males and females respectively. The numbers in each node correspond to the individual ID of each otter within their network. Lines signify association indices between each otter dyad and are scaled to indicate the strength of the associations.

**
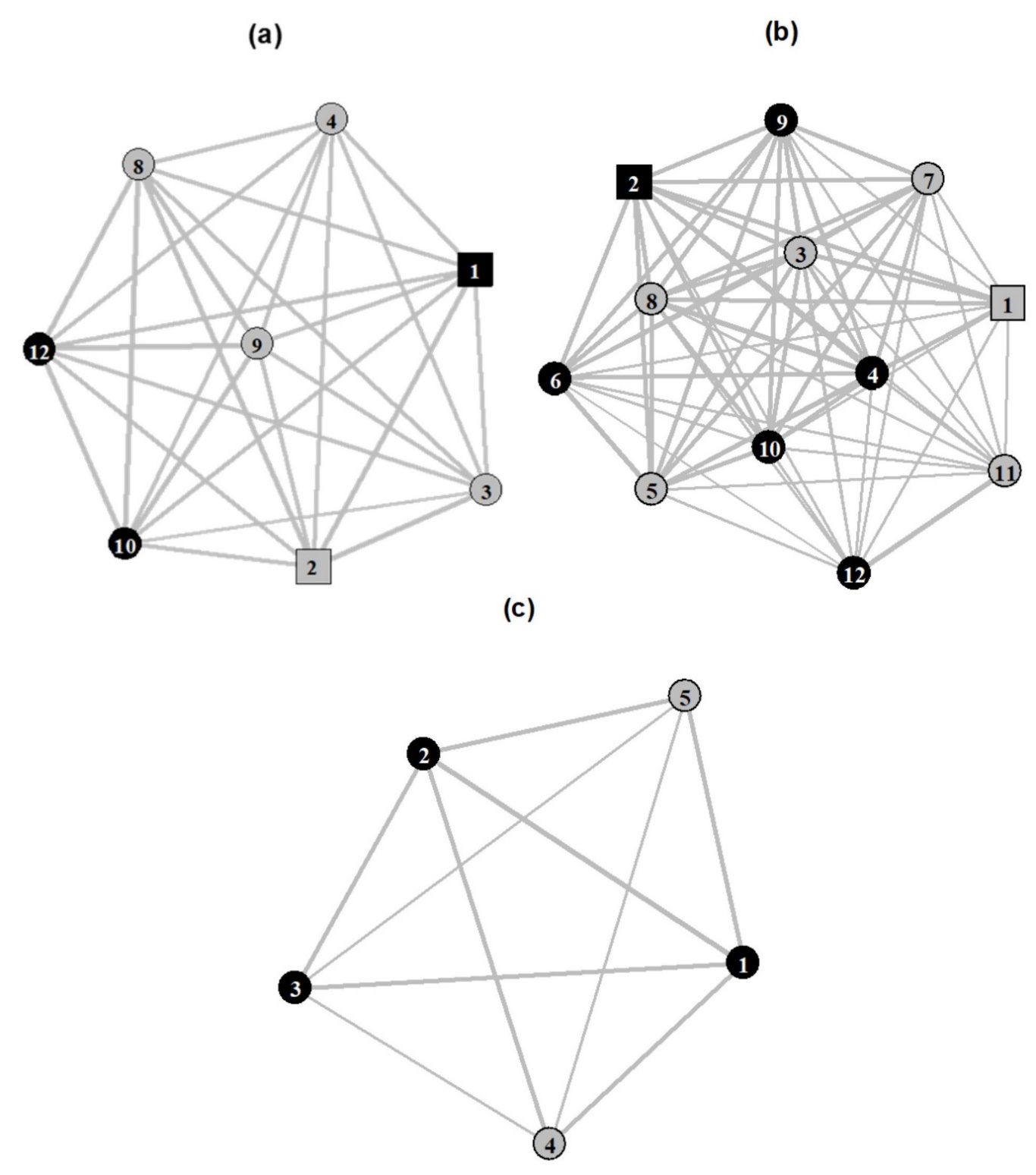
**

**Figure S2.** The Association Networks of the Asian short-clawed otter groups for the second round of the novel foraging task presentations; (a) Newquay Zoo (social differentiation coefficient; SDC: 0.41), (b) Tamar Otter and Wildlife Centre (which remained unchanged between tasks presentation rounds; SDC: 0.36), and (c) New Forest Wildlife Park (SDC: 0.58). Nodes represent group members; squares and circles represent parents and offspring respectively, and black and grey nodes represent males and females respectively. The numbers in each node correspond to the individual ID of each otter within their network. Lines signify association indices between each otter dyad and are scaled to indicate the strength of the association.
